# Emphysematous Abdominal Aortitis

**DOI:** 10.1590/0037-8682-0595-2023

**Published:** 2024-02-05

**Authors:** Elif Gündoğdu, Hilal Kırmızıgül, Serhat Demir

**Affiliations:** 1 Eskişehir Osmangazi University, Faculty of Medicine, Department of Radiology, Eskişehir, Turkey.

**FIGURE 1:**
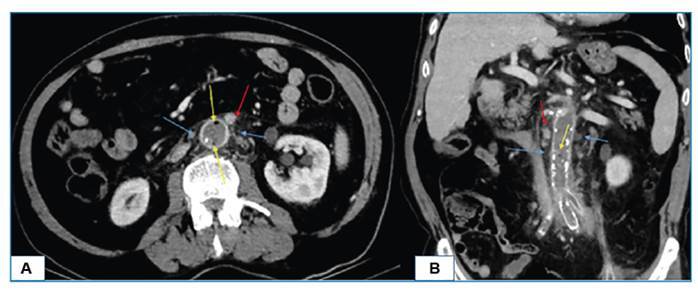
Axial **(A)** and coronal **(B)** plane abdomen CT shows occlusion of the distal abdominal aorta, perivascular fat stranding (blue arrows), vessel wall gas formation (yellow arrows), and several lymph nodes (red arrow).

A 71-year-old male patient presented to the emergency department with complaints of lower left quadrant and back pain, chills, shivering, and nausea persisting for the past two days. The patient's medical history included diabetes mellitus, coronary artery disease, and vascular intervention for aortoiliac graft placement two months ago. His body temperature was 39 °C. Laboratory tests revealed leukocytosis (13.5x103 uL) and, elevated C-reactive protein (72 mg/L) and procalcitonin (95.31 ng/mL) levels. Empirical antibiotic therapy was initiated and subsequent testing revealed a *Salmonella typhi* H antigen titer of 1/160. A computed tomography (CT) scan demonstrated occlusion of the left iliac artery and distal abdominal aorta stent, aortic wall thickening, perivascular fat stranding, vessel wall gas formation, and several lymph nodes (Figure 1). The patient was diagnosed with emphysematous aortitis, a rare yet life-threatening condition caused by gas-forming organisms^1^. The risk factors for emphysematous aortitis include atherosclerotic disease, preexisting aneurysms, diabetes mellitus, and other immunocompromised states^2^. Most reported cases of emphysematous aortitis are associated with complications of endovascular graft procedures, similar to the case presented here^3^. Because patients often present with nonspecific symptoms, imaging plays a crucial role in achieving an accurate diagnosis^2^. Therefore, CT could be a rapid and reliable diagnostic tool in such a case.
